# Can the surgical checklist reduce the risk of wrong site surgery in orthopaedics? - can the checklist help? Supporting evidence from analysis of a national patient incident reporting system

**DOI:** 10.1186/1749-799X-6-18

**Published:** 2011-04-18

**Authors:** Sukhmeet S Panesar, Douglas J Noble, Saqeb B Mirza, Bhavesh Patel, Bhupinder Mann, Mark Emerton, Kevin Cleary, Aziz Sheikh, Mohit Bhandari

**Affiliations:** 1 National Patient Safety Agency, 4-8 Maple Street, London, W1T 5HD, UK; 2 Healthcare Innovation and Policy Unit, Centre for Health Sciences, The Blizard Institute, Barts and The London School of Medicine and Dentistry Queen Mary University of London, Abernethy Building, 2 Newark Street, UK E1 2AT, London; 3 Southampton University Hospitals NHS Trust, Tremona Road, Southampton, Hampshire, SO16 6YD, UK; 4 Royal National Orthopaedic Hospital, Brockley Hill, Stanmore, Middlesex, HA7 4LP, UK; 5 Chapel Allerton Hospital and NHS Institute for Innovation and Improvement, Harehills Lane, Leeds, West Yorkshire, LS7 4SA, UK; 6 Centre for Population Health Sciences, The University of Edinburgh, 20 West Richmond Street, Edinburgh, EH8 9DX, UK; 7 Department of Orthopaedic Surgery, 293 Wellington Street North, Suite 110, McMaster University, Hamilton, Ontario, L8S4L8, Canada

## Abstract

**Background:**

Surgical procedures are now very common, with estimates ranging from 4% of the general population having an operation per annum in economically-developing countries; this rising to 8% in economically-developed countries. Whilst these surgical procedures typically result in considerable improvements to health outcomes, it is increasingly appreciated that surgery is a high risk industry. Tools developed in the aviation industry are beginning to be used to minimise the risk of errors in surgery. One such tool is the World Health Organization's (WHO) surgery checklist. The National Patient Safety Agency (NPSA) manages the largest database of patient safety incidents (PSIs) in the world, already having received over three million reports of episodes of care that could or did result in iatrogenic harm. The aim of this study was to estimate how many incidents of wrong site surgery in orthopaedics that have been reported to the NPSA could have been prevented by the WHO surgical checklist.

**Methods:**

The National Reporting and Learning Service (NRLS) database was searched between 1^st ^January 2008- 31^st ^December 2008 to identify all incidents classified as wrong site surgery in orthopaedics. These incidents were broken down into the different types of wrong site surgery. A Likert-scale from 1-5 was used to assess the preventability of these cases if the checklist was used.

**Results:**

133/316 (42%) incidents satisfied the inclusion criteria. A large proportion of cases, 183/316 were misclassified. Furthermore, there were fewer cases of actual harm [9% (12/133)] versus 'near-misses' [121/133 (91%)]. Subsequent analysis revealed a smaller proportion of 'near-misses' being prevented by the checklist than the proportion of incidents that resulted in actual harm; 18/121 [14.9% (95% CI 8.5 - 21.2%)] versus 10/12 [83.3% (95%CI 62.2 - 104.4%)] respectively. Summatively, the checklist could have been prevented 28/133 [21.1% (95%CI 14.1 - 28.0%)] patient safety incidents.

**Discussion:**

Orthopaedic surgery is a high volume specialty with major technical complexity in terms of equipment demands and staff training and familiarity. There is therefore an increased propensity for errors to occur. Wrong-site surgery still occurs in this specialty and is a potentially devastating situation for both the patient and surgeon. Despite the limitations of inclusion and reporting bias, our study highlights the need to match technical precision with patient safety. Tools such as the WHO surgical checklist can help us to achieve this.

## Background

*''In 1935, the U.S. Army Air Corps held a flight competition for airplane manufacturers vying to build its next-generation long-range bomber. In early evaluations, the Boeing plane had trounced other designs. The flight "competition," was regarded as a mere formality. With the most technically gifted test pilot in the army on board, the plane roared down the tarmac, lifted off smoothly, and climbed sharply to three hundred feet. Then it stalled, turned on one wing, and crashed in a fiery explosion. Two of the five crew members died, including the pilot. An investigation revealed that nothing mechanical had gone wrong. The pilot had forgotten to release the new locking mechanism on the elevator and rudder controls. A few months later, army pilots were convinced the plane could fly and invented something that would be used on the few planes that had been purchased... A checklist, with step-by-step checks for takeoff, flight, landing, and taxiing. With the checklist in hand, the pilots went on to fly the model (B-17) a total of 1.8 million miles through several conflicts without one accident.'' *[1]

This episode has been heralded as the key milestone in the birth of the checklist.

The delivery of healthcare is complex and hence riddled with the potential for errors due to human factors, system failures and, more commonly, a combination of the two [2]. Fortunately, many of these errors do not result in harm, but some do, often as a result of a multiplicity of minor errors co-aligning and resulting in a more serious event that results in patient harm [3]. The proliferation of epidemiological and qualitative research into medical errors has contributed to improvements in our understanding of the root causes of many of these errors [4]. Clinical outcomes, morbidity and mortality are the product of both technical and non-technical skill. Indeed analysis of error and morbidity suggest that technical failures account for only a small proportion of these. Healthcare systems are now recognized to be a series of complex interrelated Microsystems [5] where clinicians, patients and patterns of practice interact to determine the outcome [6]. It is clear that substantial aspects of clinical practice are now too complex for groups of healthcare professionals to carry out reliably from memory alone. Surgery is one such example where clinicians are faced with high levels of uncertainty in their daily work, which may impact on the quality and safety of care patients receive [7]. This understanding means that it is important for professionals (and their respective bodies) to identify and implement strategies that reduce the risk of iatrogenic harm while at the same time ensuring that optimum outcomes are most likely.

In the UK, most people will have surgery at some point in their life. Approximately 4.2 million surgical operations are carried out every year in England alone. That equates to one operation for every 12 people per year [8]. Surgery has been categorised as a very unsafe undertaking with a rate of fatal adverse events (catastrophic events per exposure) of 1 per 10,000 surgical procedures. In industrial countries, major complications occur in 3-16% of inpatient surgical procedures and permanent disability or death rates are 0.4-0.8% [1]. In trauma surgery, the rate of serious complications is substantially higher at an estimated 1 per 100 surgical exposures. By contrast, in civil aviation, railway transport and nuclear power the rate of death is less than 1 per million exposures [9].

Whilst surgical training and practice has focused on technical skills and technological advances there has been little recognition of the benefits of non-technical skills (human factors). Most of the errors that occur during surgery can be attributed to failures in these non-technical skills such as situation awareness, decision making, communication and teamwork and leadership. Other high-risk industries such as aviation and petroleum have made great progress in managing these challenges and have reduced harmful events by several orders of magnitude. They have achieved this by accepting that humans working in complex systems inevitably make errors and have provided opportunities to learn and improve performance. This insight has led to a focus on building systems that reliably deliver what is required and that identify errors that occur with built in mitigation steps that prevent errors causing harm. Central to the success of such initiatives has been an increased appreciation of the role of human factors, the value of teamwork and the principles of reliable system design. Specifically they have built formal mechanisms of communication, trained in non-technical skills and developed checklists [10].

In January 2007, the World Health Organization (WHO) began a programme aimed at improving the safety of surgical care globally. This initiative - Safe Surgery Saves Lives - identified minimum standards of surgical care that can be universally applied across countries and settings [1]. A core set of safety checks was developed in the form of a WHO Surgical Safety Checklist that can be used in any surgical setting and operating theatre environment. Each step in the checklist is simple, widely applicable, measurable, and has been shown to be associated with a reduced risk of death and major complications in a range of clinical settings. The instrument suggests three phases: Sign-in, Time-out and Sign-out. The "Sign-in" is done prior to induction of anaesthesia and includes confirmation of patient identification, consent and site-marking as well as checks for allergies, assessment of difficult airways and anticipated blood loss. "Time-out" occurs just prior to skin incision and serves to confirm the patient, site, procedure and position, the application of the surgical site infection bundle, the use of venous thromboembolism prophylaxis, the presence of the correct imaging, equipment sterility and the anticipation of any critical steps. Prior to the removal of the drapes, the "Sign-out" confirms the procedure performed and the instrument and swab counts as well as plans for post-operative management. These questions are a final check. They are intended to be usually a redundant step in the process identifying the few occasions when all other processes have failed to ensure the patient receives everything intended. This and the simple effect of knowing they are to be asked significantly improve the reliability of the clinical processes and may reduce complications by up to 50% [11].

The NPSA has instituted the NRLS database of patient safety incidents (PSIs) [12]. Running since 2003, this database is now the largest of its kind in the world, already having received over four million reports of events that caused or had the potential to cause harm [13]. Incident reporting does not reveal the true incidence or prevalence of errors, but the volume or reports gathered can provide important insights into the frequency and causes of errors, and offer opportunities to identify possible ameliorative responses [14]. Reports continue to accrue at an accelerating rate, with the database currently receiving approximately a quarter of a million reports per quarter. Data from 2008 reveal that of these, 152,017 incidents (15.5%) related to surgery and of these 32.4% (49,254 incidents) related to orthopaedics and trauma [15].

Wrong-site surgery represents a devastating event for all parties concerned. Data from the National Health Service Litigation Authority (NHSLA) in 2006 reveal that the cost of settling wrong-site surgery claims was over £1 million pounds in England alone [16]. NHSLA data also reveal that trauma and orthopaedics had the highest number of claims with 29.8% of the total compared with the next specialty, dentistry at 16.8% [17]. For example, an analysis of NHSLA data combined with the NHS records for the total number of surgical procedures carried out in the period 2006 to 2007 confirms that orthopaedic surgery has the highest rates of wrong-site surgery [18].

In the NRLS wrong site surgery is classified as any event in which surgery is performed with the 'wrong patient', 'wrong site prosthesis', 'wrong side surgery', 'wrong side marked on patient', 'wrong side block', 'wrong side marked on theatre list' and 'wrong side marked on consent form'. The aim of this study was to assess how many PSIs related to wrong site surgery occurred in orthopaedics and of those how many could have been prevented by the use of the WHO surgical checklist.

## Methods

We searched the NRLS database to identify incidents of wrong site surgery across the specialty of orthopaedics and trauma. These were incidents that occurred from 1^st ^January 2008- 31^st ^December 2008. These incidents were reported with varying degrees of harm: 'No harm', 'Low', 'Moderate' 'Severe' and 'Death' (The search strategy is available on request from the corresponding author).

To examine whether a checklist could have mitigated the wrong site surgery events, two members of the research team (BP - non-clinical and SSP - clinical) independently reviewed all PSIs relating to wrong site surgery over the stipulated time period above and electronically transcribed them to a standardized data collection sheet, The incidents were classified as:

• Wrong side marked on consent form

• Wrong patient

• Wrong site prosthesis

• Wrong side marked on patient

• Wrong side block

• Wrong side surgery

• Wrong side marked on theatre list

These were stratified further according to incidents resulting in actual harm and 'near-misses.' The likelihood of the checklist in preventing the incident was assessed using a five-point Likert scale: 1 = very unlikely, 2 = unlikely, 3 = unsure, 4 = likely and 5 = very likely. Further attempts to reduce bias were ensured through non-clinical and clinical judgement. Any disagreements were resolved through mutual discussion. Means and standard deviations were calculated for each score given by the two reviewers and a suitable graphical representation was provided.

## Results

There were 316 incidents classified as wrong site surgery in orthopaedics and trauma and reported to the NRLS in 2008. Detailed review of these incidents revealed that wrong site surgery events occurred in 133/316 cases [42.1% (95%CI 36.7-47.5%)]. There was good agreement between the two reviewers both for selecting, classifying and assessing preventability of cases (Kappa = 0.97). The remaining 183 (57.9%) cases had been misclassified and were hence excluded from further analysis. There was no evidence of any wrong site surgery in these excluded cases. These cases had information irrelevant to wrong site surgery. Some examples are given in Appendix 1.

**Figure 1:**
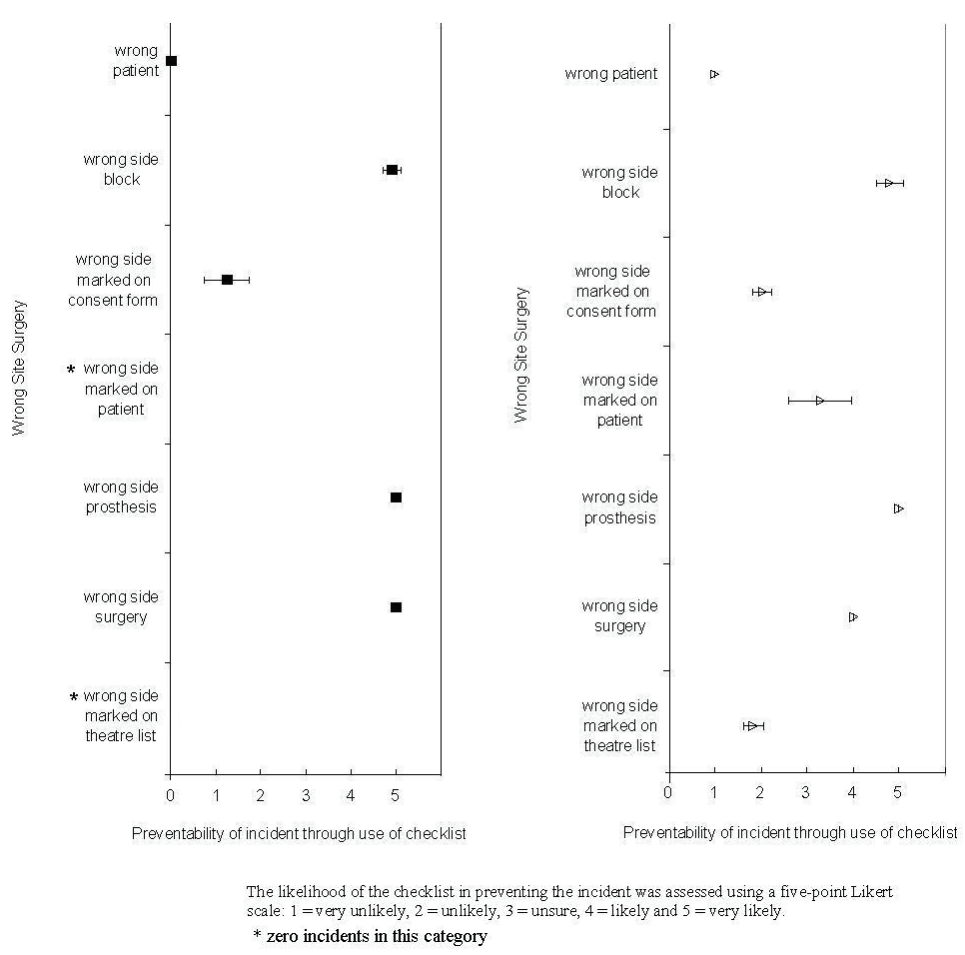
**The likelihood of the different categories of wrong site surgery being prevented through use of the checklist**.

Additional file 1 gives a sample of the different categories of wrong site surgery. The likelihood of the different categories of wrong site surgical incidents being prevented by using the checklist is shown in Figure 1.

Table 1 reveals a smaller proportion of 'near-misses' being prevented by the checklist than the proportion of incidents that resulted in actual harm; 18/121 [14.9% (95% CI 8.5 - 21.2%)] versus 10/12 [83.3% (95%CI 62.2 - 104.4%)] respectively. Summatively, the checklist could have been prevented 28/133 [21.1% (95%CI 14.1 - 28.0%)] patient safety incidents.

**Table 1:** Frequency of wrong site surgery incidents

Category of wrong site surgery	Near-misses, n (% of total)	Near-misses prevented by the checklist, n (% of individual category of wrong site surgery)	Actual harm, n (%)	Actual harm prevented by the checklist, n (% of individual category of wrong site surgery)
wrong patient	1 (0.8)	0 (0.0)	0 (0.0)	0 (0.0)
wrong side block	7 (5.8)	7 (100.0)	5 (41.7)	5 (100.0)
wrong side marked on consent form	50 (41.3)	4 (8.0)	2 (16.7)	0 (0.0)
wrong side marked on patient	9 (7.4))	4 (44.4)	0 (0.0)	0 (0.0)
wrong side prosthesis	2 (1.7)	2 (100.0)	3 (25.0)	3 (100.0)
wrong side surgery	1 (0.8)	1 (100.0)	2 (16.7)	2 (100.0)
wrong side marked on theatre list	51 (42.1)	0 (0.0)	0 (0.0)	0 (0.0)
**Total**	**121 (100.0)**	**18 (14.9)**	**12 (100.0)**	**10 (100.0)**

## Discussion

Wrong-site surgery is a potentially devastating situation for both the patient and surgeon. It does however continue to be a concern particularly in orthopaedics, despite major initiatives to address the issue, for example the "operate through your initials" campaign by the Canadian Orthopaedic Association [19], the "sign your site" initiative by the AAOS [20], the "SMaX" initiative [21] and the Royal College of Surgeons' and NPSA guidance [22]. By February 2010, all hospitals in the UK should have implemented use of the checklist. However, results of a survey indicate that more than 60% of units were evaluating or auditing whether the checklist made a difference. Only 29% of hospitals found had identified a way to record the checklist was used and having an impact [23]. A lack of robust evidence promoting the use of the checklist, briefings and debriefings can no longer be cited as a reason for slow adoption of this initiative. Two new studies by deVries EN *et al *[24] and Neily J*et al *[25] reveal that significant reductions in surgical mortality and morbidity can be made through use of checklists.

The root causes of wrong-site surgery are multifactorial. However, featuring prominently in some of the analyses include breakdown in communication between surgical team members, absence of verification in the operating theatre and of a verification checklist, incorrect marking or consent, preparation of the wrong side, incorrect draping, patient answering to the wrong name[26] and failure of a formal 'time-out' procedure [27]. In an analysis of wrong -site surgery near misses and actual occurrences, assessments in which near misses were identified that did not progress on to actual wrong-site occurrences were significantly more likely to report compliance with activities such as patient identification, preoperative reconciliation protocols, notation of surgical site on consent form, participation of the surgeon in preoperative verification and participation of all surgical team members in formal time-out procedures [28]. One of the key elements to preventing wrong-site surgery is to have multiple independent checks of critical information [29]. As we have shown, the checklist is an extremely effective tool at preventing both 'near-misses' and 'actual harm' in the following categories of wrong site surgery: wrong side block, wrong side marked on patient, wrong side prosthesis and wrong side surgery. The checklist is of limited use in ensuring correct filling in of consent forms and generation of theatre lists. Further tools such as briefings and debriefings may help in this area. The relatively high frequency of listing errors has previously been highlighted by the NPSA. For example, from 2003 to 2006 there were 855 incidents reported to the NRLS relating to erroneous details being included on operating lists [30].

Despite the fact that a large proportion of our incidents (91%) resulted in no harm, they all represent a major increase in the risk of an adverse event occurring and reveal systems with significantly degraded risk resilience. Degraded risk resilience represents a situation in which many of the barriers protecting against error have failed; there is an accident waiting to happen [31]. The capacity to defend against the potential for minor mishaps having a cumulative effect and escalating into more serious breakdowns is an essential characteristic of a reliable process. It requires a focus on the adequacy of the organisational defenses that remain in reserve and provide 'resilience' to the risk of an event escalating into a major untoward event [32,33]. It is important that our systems catch errors before they escalate and also have defensive capacity beyond this in case the events develop further, i.e. 'to survive the unforeseen' [33]. The number of 'near-misses' exceeds the cases of actual harm by a magnitude of ten, so even though only 15% of near miss incidents could have been prevented by using the checklist versus 83% of actual harm incidents, these 'near-misses' are the result of some checks or resiliency in the system. According to the Swiss-cheese model, these would be the result of certain defensive layers being intact [34].

Our study has several limitations. Analysis and interpreting data from the NRLS poses several challenges, largely due to the architecture of the NRLS. The approaches used for analyses include stratified sampling of frequently occurring incident type and free text data mining of specific topics [35]. Our search strategy may have omitted some cases of wrong site surgery. Analysis is also compromised by the lack of detail in many of the reports received and, by virtue of the fact that reports are anonymised, the lack of opportunity to easily go back to those making the reports or to case notes to identify further information [36]. It would have been useful for us to contact some of the authors of the wrong site surgery PSIs to delineate further what actually occurred. The gross under-reporting to the database has been cited as its Achilles heel [10]. This often limits the NRLS to warning, communication and detection or rare PSIs [37]. It also presents a fundamental epidemiological bias; gaining accurate data of error rates is confounded (level III/IV evidence). Whilst this is a valid criticism, it is clear that reporting is increasing as clinicians become more aware of its presence and furthermore develop confidence that there will not be any personal repercussions to making reports. Convincing clinicians of the usefulness of the data they contribute should in due course further increase the frequency and quality of reporting. Yet, it is increasingly likely that mandatory reporting offers the only viable solution to accruing reflective data. Perhaps in due course, we can assess the trends of wrong site surgery using the NRLS provided all hospitals provide accurate reports of equal quality. Although some progress has been made through the development of measures of safety and quality such as 'Never Events' trend-analysis of adverse events remains methodologically flawed [38,39].

Orthopaedic surgery is a high volume specialty with major technical complexity in terms of equipment demands and staff training and familiarity. There is therefore an increased propensity for errors to occur. Training in orthopaedic surgery focuses on technical skills. Whilst essential, this fails to recognise that surgeons cannot perform to the best of their technical ability unless in a well functioning team. Better teamwork and communication in operating theatres improves outcomes, reduces risk, improves staff well-being and mental health, reduces staff turnover and reduces delays and glitches in the surgical process. These are all improvements that will directly benefit surgeons and training. Teamwork is definable and measurable and can be improved through formal structured communication, such as checklists. Healthcare, and surgery in particular, is a team-based service yet we have ignored the experience of other high-risk industries to our patients cost. The WHO checklist and associated briefings and de-briefings are a major step forward in our approach to delivering the safe reliable care we would want for our family and to all our patients. The current state of knowledge in this field makes it professionally unacceptable to continue without using these simple yet effective tools to improve all aspects of peri-operative care.

## Conclusions

Orthopaedic surgeons take pride in their craft and there is utmost precision deployed in repairing insult to bone. Perhaps it is time, that we applied the same precision to mitigating against errors. The checklist is one such weapon in the armamentarium of the orthopaedic surgeon.

## Competing interests

The authors declare that they have no competing interests.

## Authors' contributions

SSP conceived the idea, made substantial contributions to the analysis and interpretation of the data and drafted the earlier versions of the manuscript. All authors gave final approval of the version to be published. DJN and SBM made substantial contributions to the interpretation of the data and drafted the earlier versions of the manuscript. BP made substantial contributions to the acquisition and analysis of the data and drafted the earlier versions of the manuscript. BM made substantial contributions to the interpretation of the data and drafted the earlier versions of the manuscript. ME, KC, AS, MB made substantial contributions to the interpretation of the data and revised the manuscript critically for important intellectual content. All authors read and approved the final manuscript

## Appendix 1 - some examples of misclassified incidents

'Pt admitted on 11/3/8. Not given any of her cardiac medications for about 48 hrs because they were not on the ward, including her beta - blocker . .'

'Blister noted to right heel, 2 × 1 cm, pink skin, skin intact, grade 2 sore. Left exposed and procedure three. For gel heel pad. Waterlow score amended . .'

'The patient was booked for surgery under the consultant orthopaedic surgeon. It was scheduled on the list of orthopaedic fellow on 25/3/08 and consultant anaesthetist. Patient was scheduled as the last patient on the list for left total knee replacement. Following spinal/epidural anaesthesia it was noted that the only X-ray present was for the right knees. Left knee × - rays could not be located. Decision made to cancel surgery and arranged X-ray of left knee for surgery at a later date . .'

'Found expired Warfarin tablets whilst checking TTOs for patient . .'

'Pt list admission for right total hip. Pt presented to ward with ulcer to left big toe, therefore theatre cancelled. Pt states this ulcer developed approx 1 month ago but did not contact pre - op assessment to inform them . .'

## Supplementary Material

Additional file 1 **Examples of wrong site surgery**. Click here for file
