# Supplementary material for: Can the surgical checklist reduce the risk of wrong site surgery in orthopaedics? - can the checklist help? Supporting evidence from analysis of a national patient incident reporting system
Source: J Orthop Surg Res. 2011 Apr 18;6:18. doi: 10.1186/1749-799X-6-18 (PMC3101645; doi:10.1186/1749-799X-6-18)
Supplement: Additional file 1 — Examples of wrong site surgery. [file 1749-799X-6-18-S1.DOC]

Additional file 1: Examples of wrong site surgery

***Wrong side marked on consent form***

“Patient consented for bilateral tennis elbow release , on arrival in PACU , patient had had a left tennis elbow release and injection to right elbow . Consent form had not been changed , when asked why the patient had not been consented for correct procedure the registrar ( PG ) said they could not be bothered . .”

***Wrong patient***

*“Listing forms for surgery completed in Orthopaedic Middle Grade Clinic 3.3.08 . Clinic letters typed 5.3.08 , when it became apparent that one of the listing forms had the wrong patient label on - therefore one patient listed for the wrong procedure and another not listed at all ...”*

***Wrong site prosthesis***

*“Pt came to theatre 6 to have revision left THR* [total hip replacement] *operation done . When it came to inserting the prosthesis . Consultant asked for a 36mm medium neck ceramic head , which he was shown . He checked the implant and inserted it . This was in fact the wrong size implant and the pt had to come back to theatre the following day to have a 32 , , head inserted . .”*

***Wrong side marked on patient***

*“No signature from ward staff . Section 1 and 2 on correct site surgery form . No arrow on patient to identify correct site for surgery . Backslab was only identification for correct side . .”*

***Wong side block***

*“Consultant carried out a lumbar sympathetic block on the wrong side ( right instead of left ) . Patient consented by fellow . Consent checked and patient marked by Consultant and agreed to side - but wrong side . Sedation given for procedure - after no ill effects . Discussed with patient ( still emerging from sedation ) , so needs further discussion . Medical Director informed . .”*

***Wrong side surgery***

*“MT THE ABOVE PATIENT WAS LISTED FOR A MICRODISCECTOMY . AT THE CLOSE OF PROCEDURE MR S RETURNED TO THEATRE AND INFORMED THEATRE STAFF THAT HE HAD PERFORMED THE DISCECTOMY ON THE LEFT SIDE RATHER THAN THE INTENDED RIGHT SIDE . THE PATIENT HAD TO BE REOPENED IN ORDER FOR THE DISC PROTRUSION TO BE REMOVED . MR S HAS DOCUMENTED THIS AND HAD SPOKEN TO MR T. .”*

***Wrong side marked on theatre list***

*‘‘AM operating list inaccurate . 1st patient was a left knee replacement , the operating list stated it was a right knee replacement . 2nd patient on list was a diabetic and should be have been first on list . It did not state on the operating list that the patient was diabetic ‘’*
